# The Art of Living in Australia

**Published:** 1894-09

**Authors:** 


*The Art of Living in Australia.* By PHILIP E. MUSKETT. Pp. xxix., 431. London: EYRE & SPOTTISWOODE. [1893.]

In this book the object of the author, who was lately surgeon to the Sydney Hospital, is to bring about some improvement in the extraordinary food-habits at present in vogue: it is maintained that the consumption of butcher's meat and of tea is enormously in excess of any common-sense requirements, and is paralleled nowhere else in the world; that the deep-sea fisheries are not developed; that market-gardening is deplorably neglected; and that Australian wines are not yet the national beverage of everyday life. The semi-tropical character of Australian life demands a modification of diet in accordance with existing climatic surroundings. The book contains much valuable information on food questions in relation to climate and good health, and insists on the value of home-grown wines as the most healthful and wholesome drink for Australian use.

Mrs. Wickon's three hundred cookery receipts give the book a highly practical value.

